# The implementation of a pathway and care bundle for the management of acute occlusive arterial mesenteric ischemia reduced mortality

**DOI:** 10.1097/TA.0000000000003305

**Published:** 2021-06-07

**Authors:** Matti Tolonen, Aurora Lemma, Pirkka Vikatmaa, Erno Peltola, Panu Mentula, Patrick Björkman, Ari Leppäniemi, Ville Sallinen

**Affiliations:** From the HUS Abdominal Center, Department of Abdominal Surgery (M.T., A.L., P.M., A.L., V.S.), Department of Vascular Surgery (P.V., P.B.), HUS Medical Imaging Center, Department of Radiology (E.P.), and HUS Abdominal Center, Department of Transplantation and Liver Surgery (V.S.), Helsinki University Hospital, University of Helsinki, Helsinki, Finland.

**Keywords:** Revascularization, endovascular, open abdomen

## Abstract

Acute mesenteric ischemia (AMI) is a disease with high mortality and requires a multidisciplinary approach for effective management. A pathway and care bundle were developed and implemented with the objective to reduce mortality. The aim of this retrospective comparative study was to analyze the effects of the pathway on patient management and outcome.

**METHODS:**

All consecutive patients operated in a secondary and tertiary referral center because of occlusive arterial AMI were identified between 2014 and April 2020. The pathway aimed to increase overall awareness, and hasten and improve diagnostics and management. Patients treated before implementation of the pathway (pregroup, years 2014–2017) were compared with patients treated using the pathway (postgroup, May 2018 to April 2020). Univariate and multivariate analyses were used to compare the groups.

**RESULTS:**

There were 78 patients in the pregroup and 67 patients in the postgroup with comparable baseline characteristics and disease acuity. The postgroup was more often diagnosed with contrast-enhanced computed tomography (58 [74%] vs. 63 [94%], *p* = 0.001) and had shorter mean in-hospital delay to operating room (7 hours [interquartile range, 3.5–12.5] vs. 3 hours [interquartile range, 2–11], *p* = 0.023). Revascularization was done more often in the postgroup (53 [68%] vs. 56 [84%], *p* = 0.030) especially using endovascular treatment (26 [33%] vs. 43 [64%], *p* < 0.001). Thirty-day mortality was lower in the postgroup (23 [51%] vs. 17 [25%], *p* = 0.001). Being managed in the postgroup remained as a protective factor (odds ratio, 0.32; 95% confidence interval, 0.14–0.75; *p* = 0.008) for 30-day mortality in the multivariate analysis.

**CONCLUSION:**

Implementing a pathway and care bundle resulted in enhanced regional and in-hospital awareness of AMI, more appropriate computed tomography imaging, shorter in-hospital delays, increased number of revascularizations, and, hence, lower mortality.

**LEVEL OF EVIDENCE:**

Therapeutic/Care Management, level IV.

## Abstract 2

Supplemental digital content is available in the text.

Acute mesenteric ischemia (AMI) is a notorious disease with high mortality, usually reported between 50% and 80%.^1–3^ It may have an arterial or venous etiology. The far more common arterial AMI is furthermore divided into superior mesenteric artery thromboembolism and nonocclusive mesenteric ischemia.^4^ Even though AMI is a relatively rare condition (1:1,000)^5^ in unselected emergency department population, the incidence rises exponentially with increasing age.^6^ In fact, in patients older than 75 years, the incidence of AMI has been reported higher than that of acute appendicitis.^6^ Acute mesenteric ischemia patients benefit from early assessment in a surgical unit with capabilities to definitive management.^7^ The diagnosis and management of AMI are truly multidisciplinary, requiring high index of suspicion and awareness from emergency department physicians; quick referral to a competent center; preferably computed tomography angiography with precise interpretation; capability for open, endovascular, and hybrid revascularization of the bowel; gastrointestinal surgical expertise; staged surgical approach strategies with open abdomen management; intensive care unit (ICU) management; proper medications for future risk reduction; and afterwards often nutritional competence as well as proper individualized follow-up. In addition, the early management should be carried out decisively with minimal delays irrespective of the time of the day.^1–4^ Therefore, optimal management requires a well-staffed and well-equipped hospital, preferably with nonstop access to hybrid operating rooms (ORs).^5^

Despite the fact that all the staff and equipment requirements are met in large high-level hospitals, outcomes are usually still dismal. In a focused effort to improve the management and outcome of these patients, a multidisciplinary group was established to create a pathway and care bundle to guide the management of AMI especially during out of office hours. The pathway was developed according to existing evidence, published guidelines, and expert opinions.^1–4^ In addition, the pathway was trimmed to meet the circumstances of the study hospital. The key aspects of the pathway and care bundle were elevated awareness, rapid conclusive diagnostics, and interventions in a hybrid operating room (OR) with endovascular treatment (EVT) capacity as well as minimizing delay in each of these steps irrespective of the patients’ general condition.

The aim of this study was to compare the management and outcome of patients with occlusive arterial AMI during time before and after the implementation of the pathway and care bundle.

## PATIENTS AND METHODS

### Patients and Setting

This study was conducted as a retrospective cohort study in a single academic center (Meilahti Tower Hospital, Helsinki University Hospital), which serves both as a secondary and a tertiary referral hospital covering a population of approximately 1.7 million. It is the only hospital in the area managing acute vascular surgery emergencies and has the capability to perform open, endovascular, and hybrid operations at all times. Vascular surgeons or interventional radiologists, depending on patient requirements, perform the revascularization procedures. All AMI patients in the region have been centralized to the study unit. There are several other hospitals with emergency duty services in the area. These hospitals have a large variety in access to imaging and emergency surgery services; however, all have no capabilities to perform revascularization procedures.

Patients treated because of AMI in 2014 to April 2020 were recognized using several pathways. Electronic OR database was searched by using *International Classification of Diseases, Tenth Revision*, code K55 (vascular disorders of the intestine) or Nomesco Classification of Surgical Procedures codes for procedures on mesenteric vessels (PCE17, PCF16, PCF17, PCHXX, PCJ17, PCN16, PCN17, PCP16, PCP17, PCQ16, PCQ17, and PCQ99). Radiology patient records were searched for EVT procedures performed in radiology department angio suite (Nomesco Classification of Surgical Procedures codes). In addition, hospital discharge database was searched for *International Classification of Diseases, Tenth Revision*, code K55 to find all patients who had no intervention and were deemed to palliative care after computed tomography (CT) imaging. After the identification of patients, all patients’ medical records were manually browsed. Patients without AMI, ischemic colitis, AMI with other etiology than thrombus or embolism, elective procedures for chronic AMI, and small bowel strangulation, who deemed to palliative care without an intervention, whose symptoms started in the hospital while receiving treatment to another disease, and who were managed between January to April 2018, which was the time of the creation of the pathway, were excluded. The remaining patients had AMI caused by a thrombotic or embolic arterial occlusion.

In addition, radiology department data from all of the HUS Hospital District of Helsinki and Uusimaa was searched to identify all patients imaged with an AMI-specific CT protocol for an independent analysis.

The institutional review board approved the study design, and ethics committee approval was not deemed necessary because of the observational and retrospective nature of the study.

### The Pathway and Care Bundle and Its Implementation

**Figure 1:**
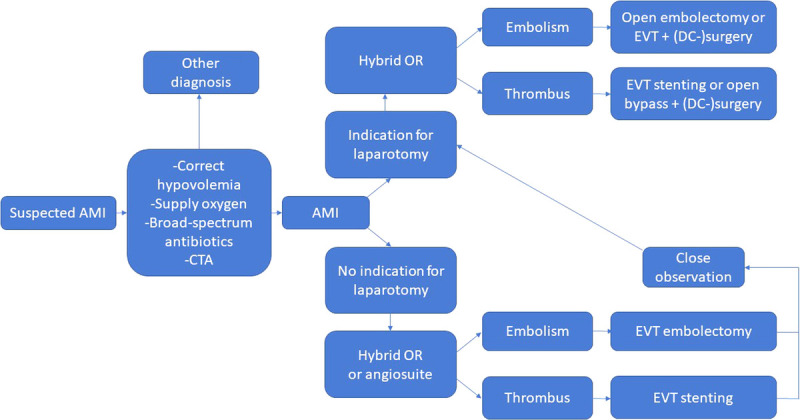
Acute mesenteric ischemia pathway. CTA, computed tomography angiography; DC, damage control.

**Figure 2:**
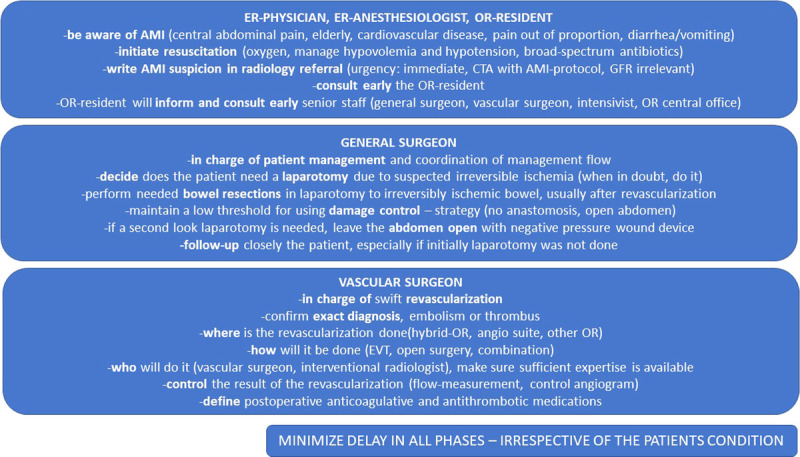
Acute mesenteric ischemia care bundle. CTA, computed tomography angiography; ER, emergency room; GFR, glomerular filtration rate.

In January 2018, a multidisciplinary group of experts was called to convene. The group consisted of several general and vascular surgeons, a radiologist, an interventional radiologist, an anesthesiologist, an intensivist, and an emergency physician. A nephrologist was also consulted. Existing evidence and published guidelines from various sources, together with expert opinions as well as understanding the local circumstances, were used as the backbone of the creation of the pathway and care bundle. Lectures by both abdominal and vascular surgeons were given, and comments from department staff were heard during the development. During the process and soon after the publication, there were several lectures given to different groups of emergency physicians, gastrointestinal and vascular surgeons, and primary care physicians and intensivists working in the area as well as multiple national congresses. The goal was to introduce and distribute the new pathway, not only inside the study hospital, but regionally to all the referring hospitals as well as nationally to raise awareness. The final pathway and care bundle was introduced in the beginning of May 2018, and its translation is presented in Figures 1 and 2. The key altered factors were raised awareness of AMI, low threshold for suspicion, immediate CT-angiography with prespecified protocol regardless of kidney function and suspicion of AMI written in the radiology referral, real-time radiology report, early involvement of senior staff members, clear division of labor between specialties, increased utilization of hybrid ORs, minimal delay access to hybrid OR or angio suite (scheduled within 2 hours), prioritizing early and effective revascularization, preferring damage-control strategy after laparotomy with deferred anastomosis and open abdomen with negative pressure wound therapy, and minimizing delays in all steps regardless of the patients’ clinical condition. The group considered that the main role of the pathway and care bundle is to increase awareness of AMI and to act as a memory list for clinicians on call who rarely manage AMI patients.

### Definitions

Comorbidities were classified according to the Charlson Comorbidity Index and the American Society of Anesthesiologist classification.^8^ Preoperative acute organ dysfunctions were classified according to the Sepsis-III guidelines.^9^ The prepathway time was 4 years, 2014 to 2017, and the postpathway time 2 years, May 2018 to April 2020.

### Statistical Analyses

Descriptive statistics for dichotomous variables are presented in number and percentage and for continuous variables in median and interquartile range. Univariate analyses for categorical variables were tested using χ^2^ test or Fischer’s exact test, where appropriate. All continuous variables were tested for normality with Shapiro-Wilk test. Univariate analysis for nonnormally distributed continuous variables was tested using Mann-Whitney *U* test. Two-tailed *p* value of <0.05 was considered significant. Multivariate binary logistic regression analysis was performed using preoperative variables that were not clearly affected by the new pathway, however avoiding multicollinearity. Goodness of fit was tested using Hosmer-Lemeshow test, and model performance was tested using Nagelkerke *R*^2^ and area under the receiver operating characteristic curve. All statistical analyses were performed using SPSS Statistics version 25 (IBM, Armonk, NY).

## RESULTS

**Figure 3:**
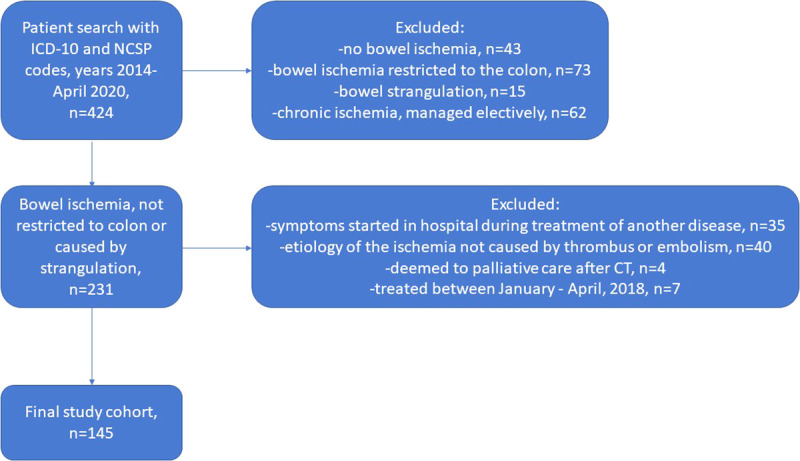
Flow chart of patient selection. ICD, International Classification of Diseases; NCSP, Nordic Classification of Surgical Procedures.

A total of 420 patients were recognized in the diagnosis- and procedure-based search, and additional 4 patients were deemed to palliative care after CT from hospital discharge database. After applying the exclusion criteria (Fig. 3), 145 patients were analyzed, 78 in the prepathway group (pregroup) and 67 in the postpathway group (postgroup). The patient characteristics are presented in Table 1. Briefly, the median age of patients was 75 years, and two thirds were female. Preoperative acute organ dysfunctions were diagnosed in nearly a third of the patients. The pregroup and postgroup were similar regarding their basic demographics, comorbidities, and several preoperative variables, such as delay, acute organ dysfunctions, and laboratory values. An exception was that the patients were a median of 4 years younger in the postgroup. The average annual incidence was 20 in the pregroup and 34 in the postgroup. More patients were referred from other hospitals in the postgroup.

**TABLE 1:** Patient Characteristics and Preoperative Data

	All, n (%)	Pregroup, n (%)	Postgroup, n (%)	*p*
Years	2014 to April 2020	2014–2017	May 2018 to April 2020	
Patients, n	145	78	67	
Age, y	75 (69–82.5)	78 (70.75–85)	74 (68–81)	0.018*
Sex, male	51 (35)	25 (32)	26 (39)	0.396
Charlson Comorbidity Index	3 (2–4.5)	3 (1–5)	3 (2–4)	0.444*
ASA classification 4–5	126 (87)	67 (86)	59 (88)	0.700
Dependent functional status	38 (26)	20 (26)	18 (27)	0.867
Symptoms >24 h before first ED	67 (46)	34 (44)	33 (49)	0.495
Referred from another hospital	81 (56)	37 (47)	44 (66)	**0.027**
Refused ICU admission	20 (14)	14 (18)	6 (9)	0.117
Acute organ dysfunctions				0.878
– No	103 (71)	55 (71)	48 (72)	
– Yes, but no shock	34 (23)	18 (23)	16 (24)	
– Shock	8 (6)	5 (6)	3 (4)	
Lactate, mmol/L	2 (1.3–3.4)	2 (1.3–3.7)	1.9 (1.3–3.2)	0.671*
CRP, mg/L	102 (26.5–251.5)	101 (25.25–229.25)	112 (27–264)	0.644*
Preoperative imaging				
No CT	7 (5)	7 (9)	0 (0)	0.015**
CT, noncontrast	17 (12)	13 (17)	4 (6)	**0.046**
CT, venous phase contrast	63 (43)	41 (53)	22 (33)	**0.017**
CT, triple phase	58 (40)	17 (22)	41 (61)	**<0.001**
CT, any contrast	121 (83)	58 (74)	63 (94)	**0.001**
AMI suspected in CT referral	60 (43)	26 (37)	34 (51)	**0.039**
Diagnostic CT in the referring center	67 (46)	30 (38)	37 (55)	**0.044**
Delay ED-OR in study hospital, hours	5.5 (2.25–12)	7 (3.5–12.5)	3 (2–11)	0.023*
Etiology embolism / thrombus	54/91 (37/63)	33/45 (42/58)	21/46 (31/69)	0.173

Continuous variables are presented as median (interquartile range). Boldface indicates statistical significance.

*Mann-Whitney *U* test.

**Fischer’s exact test (others, χ^2^).

ASA, American Society of Anesthesiologists; CRP, C-reactive protein; ED, emergency department.

Preoperative CT imaging studies show significant differences between groups in various aspects. Nearly all patients in the postgroup were diagnosed with a contrast enhanced CT scan. The use of a triphase computed tomography angiography, as recommended in the protocol, was almost tripled, and AMI was suspected significantly more often before imaging in the postgroup. In addition, diagnostic CT already performed in the referring center was more common in the postgroup, and in-hospital delay from arrival to emergency department to the beginning of intervention was cut in more than half in the postgroup (Table 1).

A specific CT protocol for suspected AMI was introduced in the beginning of 2015 (Supplemental Digital Content, Supplementary Table 1, http://links.lww.com/TA/C28). The annual average number of AMI-protocol CTs doubled in the postprotocol time. However, the rate of AMI-diagnosis remained similar in about 20%. The most common other diagnoses from suspected AMI patients were bowel obstruction or dilatation, colitis, and intra-abdominal infection or pancreatitis.

The interventions and access route to EVT and procedures done are presented in Table 2. The main differences between groups were that revascularization was done more often in the postgroup. There was a clear shift in revascularization procedures toward EVT, and more patients had their primary interventions in a hybrid OR. In the pregroup, the predominant access to EVT was the femoral artery, whereas, in the postgroup, there was more variety and close to half of EVT procedures were done using other access than the femoral artery. Of the EVT procedures, the main difference between groups was that stenting was more common in the postgroup.

**TABLE 2:** Interventions

	All (n = 145), n (%)	Pregroup (n = 78), n (%)	Postgroup (n = 67), n (%)	*p*
Vast irreversible ischemia, palliative care	16 (11)	11 (14)	5 (7)	0.203
Revascularization done	109 (75)	53 (68)	56 (84)	**0.030**
EVT	69 (48)	26 (33)	43 (64)	**<0.001**
Open embolectomy	29 (20)	19 (24)	10 (15)	0.157
Bypass surgery	24 (17)	14 (18)	10 (15)	0.625
No revascularization, active treatment	20 (14)	14 (18)	6 (9)	0.142
EVT without laparotomy	20 (14)	7 (9)	13 (19)	0.070
Laparotomy	125 (86)	71 (91)	54 (81)	0.069
Bowel resection	81 (56)	46 (59)	35 (52)	0.415
Length of bowel resection (n = 81), cm	70 (32.5–129.5)	62.5 (28.75–112.5)	73 (40–200)	0.106*
Open abdomen, laparotomy patients (n = 125)	43 (30)	20 (28)	23 (43)	0.093
Primary intervention circumstance				
– Hybrid OR	47 (32)	8 (10)	39 (58)	**<0.001**
– Conventional OR	72 (50)	57 (73)	15 (22)	**<0.001**
– Angio suite	26 (18)	13 (17)	13 (19)	0.668

EVT Access	All (n = 69), n (%)	Pregroup (n = 26), n (%)	Postgroup (n = 43), n (%)	*p*
Femoral artery	46 (67)	21 (81)	25 (58)	**0.028**
Brachial artery	9 (13)	2 (8)	7 (16)	0.466**
SMA (ROMS)	10 (14)	2 (8)	8 (19)	0.299**
Combination	4 (6)	1 (4)	3 (7)	1.000**

EVT Procedures†	All (n = 73), n (%)	Pregroup (n = 29), n (%)	Postgroup (n = 44), n (%)	*p*
Stent (+/− thrombectomy/embolectomy)	53 (73)	17 (59)	36 (82)	**0.030**
Embolectomy	15 (21)	9 (31)	6 (14)	0.084
Balloon dilatation	2 (3)	2 (7)	0 (0)	0.155**
Unsuccessful attempt	3 (4)	1 (3)	2 (5)	1.000**
Target, SMA	64 (88)	25 (86)	39 (89)	0.734
Target, celiac trunk	9 (12)	4 (14)	5 (11)	0.734**

Continuous variables are presented as median (interquartile range). Boldface indicates statistical significance.

*Mann-Whitney *U* test.

**Fischer’s exact test (others, χ^2^).

†Patient may have multiple procedures.

ROMS, retrograde open mesenteric stenting; SMA, superior mesenteric artery.

Patients in the postgroup were admitted in the ICU more often, and 30-day mortality was more than halved to 25% (Table 3). Additional subgroup mortality analyses show that significant mortality differences remain if diagnostic CT was performed in the study center and in patients who underwent laparotomy (Table 3). In patients managed without laparotomy, there were no deaths. In the multivariate binary logistic regression analysis (Table 4), preoperative acute organ dysfunctions were an independent risk factor for 30-day mortality, whereas belonging to the postgroup and ICU admission were protective factors. Nagelkerke *R*^2^ for the model was 0.36; Hosmer-Lemeshow test was 0.71, showing adequate fit; and area under the receiver operating characteristic curve was 0.81 (95% confidence interval, 0.74–0.88; *p* < 0.001).

**TABLE 3:** Outcomes

	All (n = 145), n (%)	Pregroup (n = 78), n (%)	Postgroup (n = 67), n (%)	*p*
ICU admission	59 (41)	23 (30)	36 (54)	**0.003**
ICU-free days*	22 (0–28)	10 (0–28)	23 (16–28)	0.018**
Hospital-free days*	12 (0–22)	0 (0–22)	13 (0–23)	0.039**
Mortality, 30 d	57 (39)	40 (51)	17 (25)	**0.001**
Mortality, 90 d	62 (43)	41 (53)	21 (31)	**0.010**
Mortality, 30 d, embolism (n = 54)	24 (44)	17/33 (52)	7/21 (33)	0.190
Mortality, 30 d, thrombus (n = 91)	33 (36)	23/45 (51)	10/46 (22)	**0.004**
Mortality, 30 d, diagnostic CT in the study center (n = 78)	35 (45)	28/48 (58)	7/30 (23)	**0.002**
Mortality, 30 d, diagnostic CT in the referring center (n = 67)	22 (33)	12/30 (40)	10/37 (27)	0.261
Mortality, 30 d, patients who had laparotomy (n = 125)	57 (46)	40/71 (56)	17/54 (31)	**0.006**

Continuous variables are presented as median (interquartile range). Boldface indicates statistical significance.

*Days alive and out of ICU/hospital within 28 postoperative days.

**Mann-Whitney *U* test.

**TABLE 4:** Multivariate Binary Logistic Regression of Risk Factors for 30-day Mortality

Risk Factor	Odds Ratio (95% Confidence Interval)	*p*
Preoperative acute organ dysfunctions	**8.45 (3.24–22.04)**	**<0.001**
Postgroup	**0.32 (0.14–0.75)**	**0.008**
ICU admission	**0.33 (0.13–0.84)**	**0.021**
Dependent functional status	2.23 (0.89–5.86)	0.085
Age	1.01 (0.97–1.06)	0.558
Charlson Comorbidity Index >3	1.18 (0.51–2.75)	0.694
Symptoms >24 h before first emergency department	0.93 (0.41–2.09)	0.857

Method: enter. Boldface indicates statistical significance.

## DISCUSSION

In this single-center study, the implementation of a hospital-specific multidisciplinary pathway and care bundle for the management of arterial occlusive AMI resulted in significant improvements in patient management and halved the 30-day mortality to 25% in actively managed patients, which is among the lowest reported,^1,10^ especially when considering that the study group was an unselected group of consecutive patients. The most important changes were increased awareness of AMI before imaging, more appropriate use of contrast enhanced CT imaging, shorter in-hospital delays, preferring hybrid ORs, more active revascularization mostly with increased use of EVT, and increased ICU admission rates.

The rapid diagnosis of AMI remains a significant challenge. Typically, the patients have multiple comorbidities, and there is a large variation in the symptoms. In this study, it was observed that the annual number of referred AMI patients from other hospitals more than doubled in the postgroup. Since AMI patients should be managed only in hospitals with round-the-clock revascularization capabilities, this change was desired and most likely the result of the implementation of the pathway and the efforts made to increase awareness of AMI. In addition, the proportion of correct working diagnosis before imaging in patients with subsequent occlusive arterial AMI diagnosis was higher. Still, only half of the CT referrals in the postgroup suspected AMI, a factor shown to improve diagnostic accuracy.^11^ Other half of the AMI patients were found with CT scans among acute abdominal pain patients without clinical suspicion of the diagnosis written in the radiology referral. Acute mesenteric ischemia–specific CT-protocol scans were much more common during postpathway period, which might lead into enhanced early disease identification. To clarify, the AMI-specific CT-protocol analysis was a completely separate analysis from the rest of the study.

The median in-hospital delay from arrival to the emergency department to the beginning of an intervention more than halved to 3 hours with the implementation of the pathway. We believe that there has been a change in attitudes of the hospital staff and surgeons with the implementation of the pathway. Pushing a stable patient with subtle symptoms into the hybrid OR might seem unintuitive and takes determination. It is of paramount importance to provide revascularization and bowel resection before organ dysfunctions develop, since they are the most important independent risk factor for mortality.

Rapid revascularization is possibly the most important step of the management of an AMI patient. Modern percutaneous and hybrid techniques have acted as a game changer and are recommended in all the guidelines, even though high-quality data of EVTs benefits are still lacking.^1–3^ Open embolectomy and bypass surgery are still relevant options in selected cases and after unsuccessful EVT. In this study, the use of EVT nearly doubled to two thirds of patients. The overall number of revascularized patients raised as well. The primary intervention was performed more often in a hybrid OR, which provides the best circumstances for the utilization of a full range of versatility in revascularization techniques. In addition, the variability in EVT access increased in the postgroup, indicating more decisive EVT revascularization behavior. Of note, there were six patients in the postgroup who were treated actively, but revascularization was not done. These patients were evaluated not to need a revascularization due to very distal arterial occlusion, and bowel resection only was considered sufficient. In a nonseverely ill patient, it is possible to perform an EVT revascularization only, especially if the symptoms resolve quickly after revascularization. Nearly half of the postgroup did not have a bowel resection. Therefore, there may well be a chance to safely manage considerably more patients without a laparotomy especially when considering that there were no deaths in patients who did not have laparotomy. More patients were admitted to the ICU in the postgroup. Traditionally, only patients with acute organ dysfunctions are admitted to the ICU in Finland. In the postgroup, more patients without acute organ dysfunctions were admitted. We believe that this might be due to the increased awareness of AMI patients’ management.

It must be noted that the total annual number of patients was higher in the postpathway time. The most important factor for this was the increased number of referrals from other hospitals. The referred patients had more often diagnostic imaging already done. Another factor is that the utilization of AMI-specific CT protocol doubled. A possible explanation for the mortality differences between groups is that, in the postgroup, there were less severely ill patients, referred from surrounding centers. This hypothesis was tested by comparing mortality numbers in two subgroups. The first subgroup was patients arriving with a diagnostic CT already performed in the referring hospital. The numbers in Table 3 show that the lower postgroup mortality rate was not due to referred patients. In fact, the mortality difference between pregroup and postgroup was more evident in patients diagnosed in the study hospital. The second subgroup mortality analysis was of patients who underwent laparotomy, excluding the less severely ill who underwent EVT revascularization only. The significant mortality difference remained also in this subgroup. These analyses do not support the hypothesis of different patient populations as the cause of the improved prognosis.

Several recent studies focus on comparing mortality between open and endovascular management.^12–16^ However, revascularization technique is only a single factor affecting the outcome. Bundle strategy and attempts to affect several steps in the patients pathway are less studied entities. The concept of intestinal stroke centers has been presented in France and in China.^17,18^ Indeed, the management of AMI patients is truly multidisciplinary, and prespecified protocols taking into account local resources and circumstances seem to lead into more favorable outcomes. However, these patients seek help through many different channels, and it is vital that not only the center but also the whole medical community has a high suspicion for AMI.^7^

This study has significant limitations. This is a retrospective single-center study with a limited number of patients. Thus, only associations, not causation, can be deducted from the data. There were several changes in the AMI patients’ pathway, and this study does not provide answers on which changes had the most effect on outcome. In addition, there were more referrals from surrounding hospitals in the postgroup, which may act as a confounder. However, the only differences between groups were the parameters that were attempted to enhance with the pathway and care bundle. This study sheds light on how to improve outcomes on patients with AMI. Attitudes of medical futility are unjustified; instead, multidisciplinary collaboration and building a clear pathway and care bundle through several layers of care and diagnostics seem to be of paramount importance.

## CONCLUSION

Implementing a pathway and care bundle for the management of patients with occlusive AMI resulted in lower mortality. This improvement was not accomplished with a change in a single treatment tool but with increased awareness, enhanced use of diagnostic CT imaging, shorter delays, more decisive and effective revascularization procedures, and high-level perioperative care. These results provide encouragement for centers to examine and further improve the local management protocols for AMI.

## References

[1] Björck M Koelemay M Acosta S, . Editor’s choice — management of the diseases of mesenteric arteries and veins: clinical practice guidelines of the European Society of Vascular Surgery (ESVS). *Eur J Vasc Endovasc Surg*. 2017;53:460–510. 28359440 10.1016/j.ejvs.2017.01.010

[2] Bala M Kashuk J Moore EE, . Acute mesenteric ischemia: guidelines of the World Society of Emergency Surgery. *World J Emerg Surg*. 2017;12:38. 28794797 10.1186/s13017-017-0150-5 PMC5545843

[3] Tilsed JVT Casamassima A Kurihara H, . ESTES guidelines: acute mesenteric ischaemia. *Eur J Trauma Emerg Surg*. 2016;42:253–270. 26820988 10.1007/s00068-016-0634-0 PMC4830881

[4] Lemma AN Tolonen M Vikatmaa P Mentula P Vikatmaa L Kantonen I Leppäniemi A Sallinen V. Choice of first emergency room affects the fate of patients with acute mesenteric ischaemia: the importance of referral patterns and triage. *Eur J Vasc Endovasc Surg*. 2019;57:842–849. 31126834 10.1016/j.ejvs.2019.01.002

[5] Acosta S Björck M. Modern treatment of acute mesenteric ischaemia. *Br J Surg*. 2014;101:e100–e108. 24254428 10.1002/bjs.9330

[6] Stoney RJ Cunningham CG. Acute mesenteric ischemia. *Surgery*. 1993;114:489–490. 8367801

[7] Kärkkäinen JM Lehtimäki TT Manninen H Paajanen H. Acute mesenteric ischemia is a more common cause than expected of acute abdomen in the elderly. *J Gastrointest Surg*. 2015;19:1407–1414. 25917534 10.1007/s11605-015-2830-3

[8] Charlson ME Pompei P Ales KL MacKenzie CR. A new method of classifying prognostic comorbidity in longitudinal studies: development and validation. *J Chronic Dis*. 1987;40:373–383. 3558716 10.1016/0021-9681(87)90171-8

[9] Singer M Deutschman CS Seymour CW, . The Third International Consensus Definitions for Sepsis and Septic Shock (Sepsis-3). *JAMA*. 2016;315:801–810. 26903338 10.1001/jama.2016.0287 PMC4968574

[10] Kärkkäinen JM Acosta S. Acute mesenteric ischemia (part II) — vascular and endovascular surgical approaches. *Best Pract Res Clin Gastroenterol*. 2017;31:27–38. 28395785 10.1016/j.bpg.2016.11.003

[11] Lehtimäki TT Kärkkäinen JM Saari P Manninen H Paajanen H Vanninen R. Detecting acute mesenteric ischemia in CT of the acute abdomen is dependent on clinical suspicion: review of 95 consecutive patients. *Eur J Radiol*. 2015;84:2444–2453. 26413771 10.1016/j.ejrad.2015.09.006

[12] Block TA Acosta S Björck M. Endovascular and open surgery for acute occlusion of the superior mesenteric artery. *J Vasc Surg*. 2010;52:959–966. 20620006 10.1016/j.jvs.2010.05.084

[13] Arthurs ZM Titus J Bannazadeh M Eagleton MJ Srivastava S Sarac TP Clair DG. A comparison of endovascular revascularization with traditional therapy for the treatment of acute mesenteric ischemia. *J Vasc Surg*. 2011;53:698–705. 21236616 10.1016/j.jvs.2010.09.049

[14] Murphy B Dejong CHC Winter DC. Open and endovascular management of acute mesenteric ischaemia: a systematic review. *World J Surg*. 2019;43:3224–3231. 31482344 10.1007/s00268-019-05149-x

[15] Lim S Halandras PM Bechara C Aulivola B Crisostomo P. Contemporary management of acute mesenteric ischemia in the endovascular era. *Vasc Endovascular Surg*. 2018;53:42–50. 30360689 10.1177/1538574418805228

[16] Ryer EJ Kalra M Oderich GS Duncan AA Gloviczki P Cha S Bower TC. Revascularization for acute mesenteric ischemia. *J Vasc Surg*. 2012;55:1682–1689. 22503176 10.1016/j.jvs.2011.12.017

[17] Nuzzo A Corcos O. Management of mesenteric ischemia in the era of intestinal stroke centers: The gut and lifesaving strategy. *Rev Med Interne*. 2017;38:592–602. 28259479 10.1016/j.revmed.2017.01.018

[18] Yang S Fan X Ding W Liu B Meng J Xu D He C Yu W Wu X Li J. Multidisciplinary stepwise management strategy for acute superior mesenteric venous thrombosis: an intestinal stroke center experience. *Thromb Res*. 2015;135:36–45. 25466834 10.1016/j.thromres.2014.10.018

